# Long term persistence and risk factors for anorectal symptoms following low anterior resection for rectal cancer

**DOI:** 10.1186/s12876-023-03112-8

**Published:** 2024-01-12

**Authors:** E. Koifman, M. Armoni, Y. Gorelik, A. Harbi, Y. Streltsin, S. D. Duek, R. Brun, Y. Mazor

**Affiliations:** 1 Rambam Health Care Campus, Department of Gastroenterology, Haifa, Israel; 2 Rambam Health Care Campus, Department of General Surgery, Haifa, Israel; 3 Department of Medical Neurobiology, The Hebrew University of Jerusalem, Jerusalem, Israel

**Keywords:** Anorectal manometry, Anorectal biofeedback, LARS, Quality of life

## Abstract

**Background:**

Rectal cancer is commonly treated by chemoradiation therapy, followed by the low anterior resection anal sphincter-preserving surgery, with a temporary protecting ileostomy. After reversal of the stoma a condition known as low anterior resection syndrome (LARS) can occur characterized by a combination of symptoms such as urgent bowel movements, lack of control over bowel movements, and difficulty fully emptying the bowels. These symptoms have a significant negative impact on the quality of life for individuals who have survived the cancer. Currently, there is limited available data regarding the presence, risk factors, and effects of treatment for these symptoms during long-term follow-up.

**Aims:**

To evaluate long term outcomes of low anterior resection surgery and its correlation to baseline anorectal manometry (ARM) parameters and physiotherapy with anorectal biofeedback (BF) treatment.

**Methods:**

One hundred fifteen patients (74 males, age 63 ± 11) who underwent low anterior resection surgery for rectal cancer were included in the study. Following surgery, patients were managed by surgical and oncologic team, with more symptomatic LARS patients referred for further evaluation and treatment by gastroenterologists. At follow up, patients were contacted and offered participation in a long term follow up by answering symptom severity and quality of life (QOL) questionnaires.

**Results:**

80 (70%) patients agreed to participate in the long term follow up study (median 4 years from stoma reversal, range 1–8). Mean time from surgery to stoma closure was 6 ± 4 months. At long term follow up, mean LARS score was 30 (SD 11), with 55 (69%) patients classified as major LARS (score > 30). Presence of major LARS was associated with longer time from surgery to stoma reversal (6.8 vs. 4.8 months; *p* = 0.03) and with adjuvant chemotherapy (38% vs. 8%; *p* = 0.01). Patients initially referred for ARM and BF were more likely to suffer from major LARS at long term follow up (64% vs. 16%, *p* < 0.001). In the subgroup of patients who underwent perioperative ARM (*n* = 36), higher maximal squeeze pressure, higher maximal incremental squeeze pressure and higher rectal pressure on push were all associated with better long-term outcomes of QOL parameters (*p* < 0.05 for all). 21(54%) of patients referred to ARM were treated with BF, but long term outcomes for these patients were not different from those who did not perform BF.

**Conclusions:**

A significant number of patients continue to experience severe symptoms and a decline in their quality of life even 4 years after undergoing low anterior resection surgery. Prolonged time until stoma reversal and adjuvant chemotherapy emerged as the primary risk factors for a negative prognosis. It is important to note that referring patients for anorectal physiology testing alone tended to predict poorer long-term outcomes, indicating the presence of selection bias. However, certain measurable manometric parameters could potentially aid in identifying patients who are at a higher risk of experiencing unfavorable functional outcomes. There is a critical need to enhance current treatment options for this patient group.

**Supplementary Information:**

The online version contains supplementary material available at 10.1186/s12876-023-03112-8.

## Introduction

Colorectal cancer, which includes rectal cancer, is the third most commonly diagnosed cancer globally [1]. Rectal cancer alone accounts for more than one-third of all cases. In 2020, the estimated number of rectal cancer cases in the USA was 43,340, representing 3.2% of all cancer-related deaths [2]. Furthermore, the prevalence of rectal cancer is on the rise, particularly in Western countries [3]. With advances in both surgical and adjuvant therapies for rectal cancer, there has been a decrease in the need for abdominoperineal resection with end colostomy. Instead, a preferred procedure, especially for mid and low rectal cancers, involves chemoradiation therapy followed by low anterior resection, often accompanied by a temporary protective ileostomy [4].

Nonetheless, one potential outcome of this surgical procedure is the development of a condition called low anterior resection syndrome (LARS). Individuals who experience LARS face a range of symptoms that arise after stoma reversal, including increased bowel movements, urgency, difficulty controlling bowel movements, and a sensation of incomplete evacuation. These symptoms can significantly affect patients’ quality of life [5, 6] . The prevalence of LARS is considerable, with approximately 80–90% of patients who undergo sphincter-preserving surgery experiencing different levels of symptom severity in the short term [7]. Risk factors for LARS development include a low anastomosis, temporary diverting stoma, obstructive presenting symptoms, and anastomotic complications [8]. Chemoradiotherapy, especially neoadjuvant or adjuvant radiotherapy, although decreasing the risk for cancer recurrence, causes additional damage to the motor-sensory system and has been consistently associated with a higher risk for LARS [9].

Some bowel adaptation is thought to occur by about 12 months post operatively [7]. Limited data exists on the long-term persistence of LARS symptoms in cancer survivors, or on the risk factors for these. The aims of current study, therefore, were to describe the long-term symptom outcomes of low anterior resection, to identify modifiable risk factors for the persistence of these symptoms, and specifically to evaluate the utility of perioperative anorectal physiological testing and anorectal physiotherapy treatment in these patients.

## Methods and materials

### Study population

A retrospective cohort study was performed. All consecutive patients undergoing low anterior resection surgery between 2010 and 2018 at the Rambam Health Care Campus were screened for eligibility. Exclusion criteria included active stoma at the time of follow up, active local oncologic disease or distant metastasis following surgery, or need for extended or recurrent colonic surgery. During 2019–2020, patients who met the criteria were contacted and given the opportunity to participate by responding to comprehensive questionnaires including LARS, Fecal Incontinence Severity Index (FISI), 36-Item Short Form and the Fecal Incontinence Quality of Life (FI-QOL).

**Fig. 1:**
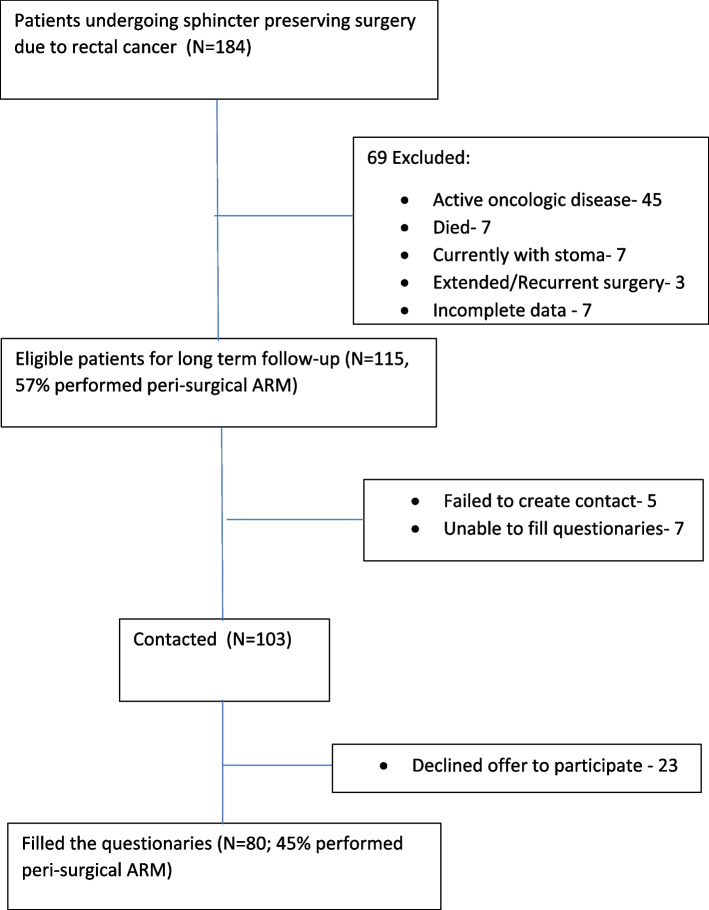
Study population patients flowchart. Abbreviation: ARM- Anorectal manometry

Study parts (Fig. 1):

1. At the retrospective part data was collected including clinical characteristics, details of primary treatment (i.e. type of surgery, chemoradiation therapy, post-surgery complications), results of perioperative anorectal physiological assessment (anorectal manometry and balloon expulsion testing) and treatment (anorectal physiotherapy with biofeedback). Coloanal anastomosis and colorectal anastomosis were defined as below and above 4 cm from the anal verge, respectively, although exact anastomotic height was not reported.
2. In the prospective phase of the study, all patients who had undergone surgery at least 1 year prior were approached and given the opportunity to participate in a long-term follow-up study. These patients were assessed using questionnaires to evaluate the severity of their symptoms and their quality of life. The severity indexes and quality of life measures obtained during the long-term follow-up were then analyzed to determine any correlations with baseline clinical characteristics, perioperative anorectal physiological testing, and anorectal physiotherapy with biofeedback treatment.

### Ethics

This study was conducted under the guidelines and approval of the Local Helsinki Committee (Approval number: 0572–17-RMB).

### Anorectal physiological testing

Patients were referred before or after stoma closure for anorectal physiological testing, including anorectal manometry (ARM) and balloon expulsion test (BET), by surgeons. For ARM, a solid-state catheter comprised of 12 circumferential sensors and a compliant balloon attached to the end was used (Medtronic, Minneapolis, USA). The catheter was connected to calibrated pressure transducers and data were displayed in digital form on a computer using ManoScan acquisition software, version 3.0 (Medtronic, Minneapolis, USA). Maximal anal sphincter resting pressure (MRP) and maximal voluntary absolute and incremental contraction squeeze pressures (MSP) were recorded. The defecation maneuver was assessed by asking the patient to ‘push down’ as if defecating, and rectal and anal pressures were recorded during the maneuver. Next, a non-latex balloon positioned in the rectal vault was inflated up to 50 ml to elicit the recto-anal sphincteric inhibitory reflex (RAIR). Gradual inflation of the same balloon by 10 ml increments up to a maximal volume of 300 ml was performed, and the intra-rectal volume required to produce an initial sensation, the first urge to evacuate and the maximum tolerated volume (MTV) were recorded. Lastly, two additional variables were documented: rectal pressure on RAIR (50 ml of air in rectal balloon) and the presence of anal slow waves (defined as cyclic and spontaneous pressure auscultations in the resting state).

Rectal BET was performed using a standard single use anorectal balloon expulsion catheter (Mississauga, ON, Canada). The procedure involved inflating the rectal balloon with 50 mL of warm water, after which the patient, seated on a private toilet, was timed to determine how long it took to expel the balloon. A balloon expulsion time exceeding 60 seconds was regarded as abnormal. This test was performed to assess the ability of the patient’s rectal muscles to expel the balloon effectively.

In cases where high pressures or very early pain (< 50 ml) were recorded in the post-surgical neo-rectum during balloon inflation, RAIR, sensory testing and balloon expulsion test were not performed in order to minimize the risk of procedure related complications such as perforation or bleeding.

### Anorectal physiotherapy treatment

For the subgroup of patients performing perioperative ARM, all were also offered pelvic floor physiotherapy and biofeedback. The physiotherapy training consisted of 30- to 60-minute once weekly sessions under the care of a single pelvic floor physiotherapist. The protocol included education regarding the anatomy of normal defecation, advice on correct toilet positioning, diaphragmatic breathing, and use of a foot stool. Instrumental biofeedback (BF) was also performed using an electromyographic (EMG) anal probe (Myomed 632x, Enraf-Nonius, Rotterdam, Netherlands) for anal muscle strengthening and endurance training. Computer assisted visual BF and verbal feedback from the therapist were used to instruct patients and improve their motor control in contraction and relaxation. In cases of weakened muscle contraction, electric stimulation was performed using the same EMG anal probe. During training of active contraction, electric stimulation to the anal sphincter was given to increase muscle strength and endurance. Patients were instructed to continue practicing at home with anal sphincter and pelvic floor exercises for relaxation, muscle squeezes and the evacuation techniques learned during the treatment sessions.

### Questionnaires at long term follow up

Questionnaires included: LARS score, Fecal Incontinence Severity Index, 36-Item Short Form and the Fecal Incontinence Quality of Life (FI-QOL). The LARS score is a validated questionnaire in multiple languages, although without specific validation for Hebrew translation. It consists of five items that are specifically designed to assess bowel function following sphincter-preserving surgery for rectal cancer [10]. The questionnaire evaluates the presence and severity of various symptoms, including flatus incontinence, liquid stool incontinence, frequency of bowel movements, clustering of stools, and urgency. The total score ranges from 0 to 42, with scores between 0 and 20 indicating no LARS, scores between 21 and 29 indicating minor LARS, and scores between 30 and 42 indicating major LARS [10, 11]. Fecal incontinence severity index (FISI) was used as a more specific measure of incontinence severity [12]. Patients were also requested to report the number of bowel movements per day and to describe their stool consistency according to the Bristol Stool Form Scale. Quality of life was assessed by two questionnaires. The Medical Outcome Study (MOS) 36-Item Short Form Health Survey (SF-36) was used as a nonspecific general health evaluation of quality of life [13]. The Fecal Incontinence Quality of Life (FI-QOL) questionnaire was used as a more specific symptom-related quality of life questionnaire [14].

### Statistical analysis

Statistical analysis was performed Using R 4.0.5 (R foundation for statistical computing). Medians and interquartile ranges, and absolute numbers and percentages were used to describe continuous and categorical variables respectively. Chi-square test was performed to compare categorical variables and Mann-Whitney U test was performed to compare continuous variables. The strength of the relationship between two quantitative measures was estimated by calculating Pearson’s r correlation coefficient. Correlation strength was evaluated as moderate at an r value between + 0.5 to + 0.7 or between − 0.7 to − 0.5 and strong at a value greater than + 0.7 or smaller than − 0.7. Multivariable logistic regression was used to assess the adjusted association between several factors both clinically important and that significantly differed on univariate analysis, and major LARS. All statistical tests were two-tailed and a *p* value less than 0.05 was considered statistically significant.

## Results

### Baseline characteristics

Among 184 patients undergoing sphincter preserving surgery for rectal cancer from 2010 to 2018, 115 patients were included in the study. Reasons for exclusion are described in Fig. 1. Baseline characteristics of patients included are described in Table 1. Tumor location was in the lower rectum (< 8 cm from the anal verge) in 68 (59%) of cases, with an average distance of 6.9 ± 3.1 cm from the anal verge. 109 (92%) patients received neoadjuvant chemoradiation before surgery and a single patient underwent only chemotherapy. All patients underwent a low anterior resection with total meso-rectal excision (TME), with coloanal anastomosis performed in 38 (33%) of patients. A temporary protective ileostomy was performed in 110 (96%) patients. Surgical staging included 40 cases at stage 0/I, 38 cases at stage II, 36 cases at stage III and one case at stage IV. 23 (20%) patients had postoperative complications, most commonly anorectal strictures (14 patients). Other complications included small bowel obstruction (7 patients), pelvic abscess or anorectal fistula (2 patients) and impotence (2 patients). Thirty three (30%) patients received post-surgical adjuvant chemotherapy. Stoma reversal was performed following a normal rectal examination, rectoscopy and gastrografin enema, an average of 6 ± 4 months from initial surgery.

**Table 1:** Baseline characteristics of patients undergoing low anterior resection (*n* = 115)

Age- mean, years (SD)	63 (11)
Gender (M: F)	73:42
Tumor distance from anal verge - mean, cm (SD)	6.9 (3.1)
Neoadjuvant chemoradiotherapy -n (%)	109^a^ (92%)
Adjuvant chemotherapy – n (%)	33 (29%)
Type of anastomosis	
Colorectal anastomosis– n (%)	77 (67%)
Coloanal anastomosis– n (%)	38 (33%)
Temporary protective ileostomy– n (%)	110 (96%)
Post-surgery complication (including pelvic abscess, anorectal fistula, and strictures) - n (%)	23 (20%)
Tumor Stage (TNM) at surgery	
0 or 1 n (%)	40 (35%)
2– n (%)	38 (33%)
3– n (%)	36 (31%)
4– n (%)	1 (1%)
Time to stoma closure- mean, months (SD)	6 (4)

^a^1 patient received chemotherapy alone

### Perioperative anorectal physiological testing and physiotherapy

Sixty-five (57%) patients underwent anorectal manometry (ARM) following surgery, 45% of them before stoma closure. Median time between surgery to ARM was 10 months (range 2–82). Anal resting pressure was low (< 68 mmHg) in 63% of patients and anal absolute squeeze pressure was low (< 100 mmHg) in 23% of patients. In 47% of patients a paradoxical contraction or absent anal relaxation were recorded on push maneuver. In 41 (63%) patients rectal sensation and/or BET were not fully performed due to high pressures in the neo-rectum or early pain. Among 24 patients who performed BET, evacuation time was abnormal (> 60 seconds) in 13 (54%) of them. No complications were noted.

Compared to colorectal anastomosis, patients who underwent coloanal anastomosis were more likely to display a low resting pressure (77% vs. 52%, *p* = 0.04) and absence of RAIR (72% vs. 25%, *p* < 0.01). Compared to patients with no post-surgery complications, patients who suffered from post-surgery complications were more likely to display a low anal squeeze pressure (33% vs. 74%, respectively, *p* = 0.05) and absence of RAIR (14% vs. 40%, respectively, *p* < 0.01). Time between surgery to stoma closure or time between surgery to ARM showed no effect on ARM results. Physiology results before or after stoma closure showed no significant difference in resting or squeeze pressures. Similarly, push maneuver dynamics did not differ before compared to after stoma closure.

24 (37%) patients underwent perioperative anorectal physiotherapy (median 4 sessions, range 1–12), 32% of them before stoma closure. Anal electric stimulation (ES) was performed in 23 (92%) of these patients. Patients with lower anal squeeze pressure, lower first sensation and lower MTV where more likely to be referred for treatment (Supplementary Table 1).

### Long term follow up

Eighty (70%) patients completed the long term follow-up questionnaires. The median time of follow-up was 4 years from stoma reversal (IQR 2–5 years). Mean time from surgery to stoma closure for these patients was 6 ± 4 months. There were no differences in baseline parameters between these patients and patients not included in the follow-up analysis (*N* = 35) (Supplementary Table 2). At long term follow up, median LARS score was 36 (IQR 26–39), with 55 (69%) patients classified as major LARS (score > 30). Other long term outcomes are summarized in Table 2.

**Table 2:** Long term outcomes (*n* = 80)

	Median [IQR]	N (%)
Time to follow up – years	4.0 [2.0, 5.0]	
LARS score (range 0–42)	36.5 [26.2,39,5]	
LARS – categorized		
Mild		16 (20%)
Moderate		9 (11%)
Major		55 (69%)
FISI (range 0–61)	23.0 [11.0, 42,2]	
SF36 (range 0–100)		
Overall average	58.3 [36.0, 81.2]	
Physical function	65.0 [30.0, 90.0]	
Social function	62.5 [25.0, 100.0]	
Role limitation – physical	25.0 [0.0, 100.0]	
Role limitation – emotional	66.7 [0.0, 100.0]	
Energy/fatigue	45.0 [35.0, 60.0]	
Emotional well being	64.0 [48.0, 76.0]	
Pain	67.5 [33.8,90.0]	
General health	55.0 [40.0, 75.0]	
FIQOL (range 0–5)		
Lifestyle	2.6 [1.6, 3.8]	
Coping/behaviour	1.8 [1.3, 3.1]	
Depression/self-perception	3.0 [1.9, 3.9]	
Embarrassment	2.3 [1.3, 3.3]	

*LARS* Lower Anterior Resection Syndrome, *FISI* Fecal Incontinence Severity Index, *SF36* 36-Item Short Form Health Survey, *FIQOL* Fecal Incontinence Quality of Life

#### Predictors of long term LARS outcomes

##### Clinical and surgical predictors of long term outcomes

Patients with major LARS at long term were compared with patients with non-major LARS (Table 3). Presence of major LARS was associated with a longer time delay from primary surgery to stoma reversal (6.8 vs. 4.8 months, *p* = 0.03) and with undergoing adjuvant chemotherapy (38% vs. 8%; *p* = 0.01). There was a borderline significant association between adjuvant chemotherapy and delay in stoma closure (median 4 vs. 6 months; *p* = 0.05), although a multi-variate analysis incorporating these two predictors did not reveal either as independent risk factors. Patients referred for perioperative physiological testing by the surgical team were more likely to still suffer from major LARS at long term follow up (64% vs. 16%, *p* < 0.001). Timing of ARM before or after stoma closure or time following surgery was not associated with outcome measures (median 11 months [IQR: 6–23] in major LARS vs. 10 months [IQR: 5–18] in the non-major LARS group). No significant differences in outcomes was observed regarding gender, neoadjuvant radiotherapy or perioperative complications (Table 3). On multivariable analysis, incorporating coloanal anastomosis and protective ileostomy as independent variables, colorectal anastomosis was associated with a reduced risk of major LARS at follow up (OR 0.22; 95% CI 0.03, 0.88; *p* = 0.03). Additionally, for each additional month of delay in closing the temporary stoma, there was a trend for increased risk for long term LARS, although not reaching statistical significance (OR 1.10; 95% CI 0.93, 1.38; *p* = 0.07).

**Table 3:** Comparison of long-term outcomes following low anterior resection: patients with major (scores ≥30) low anterior resection syndrome (LARS) vs non-major (scores < 30) LARS

	Major LARS *N* = 55	Non-major LARS *N* = 25	*P* value for difference
Age- years; mean (SD)	61.4 (12.3)	65.3 (9.9)	NS
Distance of tumor from anal verge-cm; mean(SD)	6.5 (3.3)	6.9 (3.5)	NS
Time to stoma closure –months; mean(SD)	6.8 (4.6)	4.8 (2.8)	0.03
Average time of follow-up- years; mean(SD)	3.7 (1.8)	4.4 (1.5)	NS
Gender – male; n (%)	37 (67%)	17 (68%)	NS
Pathological staging at surgery; n(%)			
Stage 0	10 (18%)	9 (36%)	NS
Stage 1	7 (12.7%)	4 (16%)	
Stage 2	18 (33%)	4 (16%)	
Stage 3	19 (35%)	8 (32%)	
Stage 4	1 (2%)	0 (0%)	
Neo-adjuvant chemoradiation therapy; n (%)	52 (94.5)^a^	21 (84)	NS
Patients with colo-anal anastomosis; n (%)	23 (42)	6 (24)	0.09
Protective ileostomy; n (%)	53 (96.4)	23 (92)	NS
Perioperative anastomotic dehiscence/pelvic abscess; n(%)	2 (3.6)	0 (0)	NS
Adjuvant chemotherapy; n (%)	21 (38)	2 (8)	0.01
Anal/rectal stricture following surgery; n (%)	6 (10.9)	2 (8)	NS
Referred for anorectal manometry; n (%)	35 (64)	4 (16)	< 0.001
Referred for anorectal biofeedback; n (%)	22 (40)	2 (8)	0.003

*SD* Standard deviation, *NS* non significant

^a^1 patient received chemotherapy alone

##### Perioperative anorectal physiological testing results as predictors of long term outcomes

Thirty nine patients in the long term follow up cohort had been referred for perioperative physiology testing. Two of these patients failed to perform ARM and one patient performed ARM only after 80 months, and these 3 patients were excluded. In the remaining patients who underwent testing and were available for long term follow up (*n* = 36; 45%), higher squeeze (absolute or incremental) anal pressures and higher rectal pressures on push were all associated with better quality of life as measured by FIQOL questionnaire (*p* < 0.05 for all, Table 4).

**Table 4:** Correlation between perioperative anorectal manometry results and long term follow up questionnaire scores (*n*=36)

	LARS score		FISI score		SF36 Overall Average		FIQOL Lifestyle	
	correlation	*P* value	correlation	*P* value	correlation	*P* value	correlation	*P* value
MRP	-0.088	0.61	+0.179	0.3	+0.24	0.16	+0.036	0.84
MSP	-0.254	0.13	+0.013	0.94	+0.09	0.61	+0.457	**0.01**
MISP	-0.198	0.25	-0.01	0.96	-0.011	0.95	+0.469	**0.01**
Rectal Pressure on Push	-0.275	0.11	-0.269	0.12	+0.4	0.02	+0.488	**0.01**
Rectal Pressure on RAIR 50 ml	+0.074	0.71	+0.079	0.7	-0.064	0.76	-0.154	0.452
First Sensation^a^	-0.026	0.92	-0.144	0.56	+0.199	0.43	+0.009	0.97
Urge^a^	+0.001	0.99	-0.188	0.5	+0.254	0.38	-0.032	0.14
MTV^a^	-0.333	0.27	-0.283	0.35	-0.06	0.85	-0.138	0.67

*MRP* maximal resting pressure, *MSP* maximal squeeze pressure, *RAIR* Rectoanal inhibitory reflex, *MISP* Maximal Incremental Squeeze Pressure, *First sensation* First rectal sensation threshold, *Urge* Defecation urge sensation threshold, *MTV* Maximal tolerated volume

^a^Procedures performed in 42% of patients

Following referral for testing, 19 (53%) of these 39 patients were treated with anorectal physiotherapy (median 4 sessions, range 1–12), 32% of them before stoma closure. Long term outcomes for these patients were poor, similar to patients referred to ARM but who did not perform BF (major LARS in 94% vs 95%, respectively; p = NS; Table 5).

**Table 5:** Association between biofeedback treatment and long term functional outcomes and quality of life in patients referred for perioperative anorectal biofeedback (*n* = 39)

	Anorectal biofeedback *N* = 21	No anorectal biofeedback *N* = 18	*P* value
Major LARS – n (%)	20/21 (95%)	17/18 (94%)	NS
LARS score – mean (SD)	38.5 (3.4)	35.9 (6.6)	NS
FISI score – mean (SD)	37.2 (15.7)	35.1 (15.6)	NS
SF-36 score – mean (SD)	52.9 (15.8)	52.9 (23.6)	NS
FI QOL Lifestyle – mean (SD)	1.9 (0.9)	1.9 (0.9)	NS
FI QOL Coping – mean (SD)	1.7 (0.8)	1.7 (0.8)	NS
FI QOL Self – mean (SD)	2.3 (0.8)	2.4 (0.9)	NS
FI QOL Embarrassment – mean (SD)	1.8 (0.9)	1.9 (0.9)	NS

*SF-36* Short form health state questionnaire, *FI-QOL* fecal incontinence quality of life, *LARS* Low anterior resection ssyndrome score, *FISI* Fecal incontinence severity index

## Discussion

Our study aimed to investigate long term anorectal symptoms and their impact on quality of life in rectal cancer survivors following low anterior resection, and correlation of these symptoms to baseline anorectal manometry (ARM) parameters and physiotherapy with anorectal biofeedback (BF). The main finding of the study is the long term persistence of severe symptoms and impairment in quality of life. Severity score results at a median of 4 years follow up showed 69% of patients still reporting symptoms of major LARS, and 84% of patients reporting some degree of fecal incontinence. The prevalence of LARS in the literature is wide, reporting a range of 25 to 80% of post-surgical patients [8, 15–18]. This difference might be explained by the different prevalence of suspected risk factors for LARS between the studies. For example, in the study of Sturiale et al. [17], only 20% of patients reported symptoms of major LARS following low anterior resection. In this study, 42% of patients did not have a temporary stoma constructed, and only 44% of patients received neoadjuvant therapy. Similarly, none of the patients in the study by Ekkart et al. [8] received neoadjuvant therapy and only 41% had a temporary stoma, resulting in only 18% prevalence of major LARS. This is contrast to our cohort, where almost all patients received neoadjuvant chemoradiation therapy and had a temporary protective stoma, probably resulting in a higher risk for developing LARS.

When symptoms of LARS do appear, they are unfortunately often long lasting. In a large retrospective study conducted on patients who underwent curative resection for rectal cancer in Denmark between 2001 and 2007, 41% of patients still experienced symptoms of major LARS at a mean follow-up of 54 months, while no association was found between major LARS and the time since surgery [16]. Our results show even worse long term outcomes, possibly again relating to our higher prevalence of baseline risk factors.

In patients with a temporary stoma, we show that a delay in reversal surgery was associated with worse quality of life at long term follow up. This effect of prolonged intervals between surgery to stoma closure shown in our study is consistent with the results of a recent meta-analysis [19]. While the construction of a temporary stoma is recommended in most guidelines as it reduces the rate of anastomotic leakage and reoperations, the optimal timing of stoma closure ranges widely and is not yet clearly defined [20, 21]. Some studies recommend early closure of the stoma to reduce morbidity, even as early as 2 weeks following initial surgery, while others have shown that stoma closure earlier than 3 months after initial surgery was associated with increased morbidity [21]. Our results provide another incentive for early rather than later closure of the stoma. Multiple factors may explain delay in stoma closure, including patient-, surgical- and oncological-related factors. Due to our study design we could not assess for all factors, but we do show that adjuvant chemotherapy by itself was also associated with long term major LARS. This finding goes in line with recent meta-analysis by Ye and colleagues [22]. Whether postponing stoma reversal until chemotherapy completion or chemotherapy itself are the major risk factors remains unanswered.

As expected, our study findings confirmed the significance of anastomosis height in relation to postoperative outcomes. Another recent meta-analysis revealed that a lower tumor height, resulting in a lower anastomotic height, was linked to a higher likelihood of developing LARS after surgery; specifically, individuals with less than 4 cm of remnant rectum had a 46% risk of experiencing major LARS, whereas those with 4 cm or more of remnant rectum had a lower risk of 10% for major LARS [23]. Our study revealed even more unfavorable outcomes in the long term for this patient group. Other patient characteristics (age, gender, tumor stage, surgery complications etc.) were not associated with long term symptom severity. Our observation regarding similar results achieved between the genders are in line again with Ye et al. recent meta-analysis [22]. We do note though that these finding in our cohort might be explained by the high overall rates of patients suffering from severe symptoms of LARS, making it difficult to perform an accurate analysis of other factors affecting these symptoms.

In our study referral for per-operative physiological testing by itself was associated with worse long term results, probably reflecting a referral bias. The ability of anorectal physiological testing results to predict long term outcomes of patients is debated [24–28]. A subset of our patients performed perioperative ARM and BET, and some parameters were shown to predict long term impact of bowel dysfunction on quality of life: higher absolute and increment squeeze pressures were found to correlate with less severe quality of life measures on follow up. Nevertheless, the clinical implications of these findings might be limited, as definition of normal and abnormal values is problematic, and categorizing patients as “poor sphincter function” might be difficult [24]. About 40% of patients in our cohort suffered from major LARS despite having normal anal sphincter function on anorectal manometry. While some previous studies have shown the benefits of ARM in evaluating fecal incontinence and/or constipation due to non-surgical etiologies [25], studies evaluating the correlation between ARM and symptoms of bowel dysfunction in surgical patients vary in their results. Dulskas et al. [26] showed no correlation between severity of incontinence and results of anorectal manometry following surgert. On the other hand, Inhát et al. [27] showed that patients with major LARS displayed significantly lower resting pressures and sensation thresholds, compared to patients with no LARS or minor LARS. Similarly, Matzel et al. [ 28] showed maximal tolerable volume and neorectal compliance were significantly correlated to incontinence severity following LAR. Thus, it seems that although ARM might have some role in predicting quality of life or symptom severity following stoma reversal, the clinical use of this data might be problematic.

Anorectal physiotherapy treatment had no effect on long term outcomes in our study. This contrasts with previous studies [29–32]. For example, Bartlett et al. [29] showed an improvement in continence and symptom related quality of life in post-surgical patients undergoing BF treatment. Reduction in scores of severity indexes was demonstrated following treatment in the studies by Kim et al. [30] and Liang et al. [31], as well as improvement of anorectal physiological function on anorectal manometry. Similar effect of BF on symptoms was shown by Pucciani et al. [32], yet no change in physiology testing results was seen in this study. Several reasons might explain the minimal effect of physiotherapy seen in our study. First, as no baseline measurements were made in our study prior to treatment, a before-after analysis of symptoms improvement could not be made. Second, measurements of outcomes in the aforementioned studies, as well as in other studies evaluating BF treatment, were made shortly after the final treatment session. As shown by Mazor et al. [33], benefits of BF in patients with fecal incontinence due to non-surgical etiology waned in about a third of the patients at a median of 7 year long-term follow-up. Moreover, at long-term follow-up, improvements in patients’ quality of life measures following BF were no longer evident. This declining trend might explain the minimal effect of BF on long term outcomes seen in our study. Lastly, in our study, baseline anorectal physiological testing results in patients who performed physiotherapy were worse than in patients that were not treated. As ARM was performed before physiotherapy, this suggests that patients who presented with a more impaired anorectal function were more likely to be referred for additional therapy such as physiotherapy, reflecting a selection bias.

Due to the retrospective design of our study, it is challenging to establish definitive diagnostic and therapeutic conclusions. Still, an important finding of the current study is the inconsistencies in diagnosis and treatment of patients following low anterior resection, as evident by the time differences in stoma closure and referral to physiotherapy, performance of anorectal physiological testing either before or after stoma closure, and the variable number of physiotherapy sessions. Current treatment options for LARS are symptom based, using existing options for non-surgical patients with fecal incontinence, fecal urgency, and rectal evacuatory disorders. Moreover, sacral neuromodulation, a relatively recent treatment option for patients with LARS, was not available to our patients during the study time-frame. Our results emphasize the strong need for prospective studies examining a well-established protocol and evaluating patients’ symptoms, quality of life and anorectal physiology results before and after treatment, including a long term follow up arm.

In summary, our study provides evidence for the long-term persistence of major LARS symptoms and a decline in quality of life among the majority of patients who underwent low anterior resection surgery. Longer intervals between surgery and stoma closure and adjuvant chemotherapy and were found to be associated with an increased risk of LARS severity, emphasizing the importance of carefully considering the timing of stoma reversal surgery. Additionally, poor anal sphincter function, as determined by anorectal manometry, either before or after stoma closure, was predictive of a lower quality of life. Our findings suggest that the studied physiotherapy treatment protocol offers minimal long-term benefit, at least for the more severely affected patients. There is a critical need for improvement in current treatment options, including the use of a more comprehensive anorectal bowel function protocol and/or sacral neuromodulation, to better address suffering in this patient population.

### Supplementary Information

**Additional file 1.**

## Data Availability

We attach the our raw data to the manuscript.
