# Supplementary material for: Long term persistence and risk factors for anorectal symptoms following low anterior resection for rectal cancer
Source: BMC Gastroenterol. 2024 Jan 12;24:31. doi: 10.1186/s12876-023-03112-8 (PMC10787434; doi:10.1186/s12876-023-03112-8)
Supplement: Supplementary file 1 — Additional file 1. [file 12876_2023_3112_MOESM1_ESM.docx]

| **Supplementary Table 1:** Association between peri-surgical biofeedback treatment and anorectal manometry (n=65). | | | | |
| --- | --- | --- | --- | --- |
|  | All patients  N=65 | Biofeedback patients  N=24 | No biofeedback patients  N=41 | P value |
| MRP- mean ± SD | 61 ± 35 | 52 ± 28 | 66 ± 38 | NS |
| MSP- mean ± SD | 151 ± 73 | 138 ± 83 | 158 ± 66 | 0.05 |
| RAIR- absent (%) | 29(48%) | 13(59%) | 16(41%) | NS |
| ARBD-absent (%) | 29(47%) | 12(57%) | 17(42%) | NS |
| BET- unable to expel (%)* | 13(54%) | 4(50%) | 9(56%) | NS |
| First Sensation - mean ± SD* | 43 ± 23 | 34 ± 21 | 48 ± 23 | 0.03 |
| Urge- mean ± SD* | 62 ± 28 | 50 ± 25 | 69 ± 29 | NS |
| MTV- mean ± SD* | 84 ± 32 | 66 ± 33 | 98 ± 24 | 0.02 |
| MRP=maximal resting pressure, MSP= maximal squeeze pressure, RAIR- Rectoanal inhibitory reflex, ARBD= anal relaxation on bear down manoeuvre, BET= balloon expulsion time, First sensation= First rectal sensation threshold, Urge= Defecation urge sensation threshold, MTV= Maximal tolerated volume.  *Procedures performed in 42% of patients | | | | |

**Supplementary table 2: Baseline data for long term cohort (n=80) compared to non-responders/unreachable (n=35).**

|  | N=80  Mean (SD);  N (%) | N=35  Mean (SD);  N (%) | P value for difference |
| --- | --- | --- | --- |
| Age -yrs | 63 (12) | 64 (10) | 0.30 |
| Gender – male | 54 (68%) | 20 (57%) | 0.29 |
| Distance of tumor from anal verge-cm | 6.7 (3.3) | 7.6 (2.7) | 0.12 |
| Staging – 0 | 19 (24%) | 8 (24%) | 0.26 |
| 1 | 11 (14%) | 2 (6%) |  |
| 2 | 22 (28%) | 16 (47%) |  |
| 3 | 27 (34%) | 8 (24%) |  |
| 4 | 1 (1 %) | 0 (0%) |  |
| Radiation therapy – yes | 72 (90%) | 33 (94%) | 0.36 |
| Chemotherapy – yes | 73 (91%) | 33 (94%) | 0.45 |
| Anastomosis type – coloanal | 29 (36%) | 9 (26%) | 0.29 |
| Colo-rectal | 51 (64%) | 26 (74%) |  |
| Protective ileostomy – yes | 76 (95%) | 34 (97%) | 0.52 |
| Anal/rectal stricture or abscess following surgery – yes | 10 (13%) | 2 (6%) | 0.47 |
| Time between surgery and stoma closure - months | 6.2 (4.2) | 6.1 (3.4) | 0.66 |
| Referred for ARM - yes | 39 (49%) | 19 (54%) | 0.59 |
| Time between surgery and ARM – months | 16.1 (16.1) | 14.6(11.9) | 0.45 |
| Anal slow waves – yes | 14/35 (40%) | 6/19 (32%) | 0.56 |
| Maximal anal resting pressure – mmHg (n=56 ) | 74.7 (31.2) | 89.5 (33.9) | 0.50 |
| Maximal anal resting pressure – high | 7/37 (19%) | 6/19 (32%) | 0.33 |
| Maximal anal resting pressure – low | 13/37 (35%) | 3/19 (16%) | 0.21 |
| Maximal anal squeeze pressure – mmHg (n=56) | 181.0 (77.9) | 199.9 (75.5) | 0.91 |
| Maximal anal squeeze pressure - low | 14/37 (38%) | 5/19 (26%) | 0.55 |
| Maximal anal squeeze pressure increment -mmHg (n=55) | 120.4 (82.5) | 125.3 (69.0) | 0.46 |
| Maximal anal squeeze pressure increment – low | 10/36 (28%) | 4/19 (21%) | 0.75 |
| Rectal pressure on push – mmHg (n=55) | 43.9 (23.9) | 41.1 (24.1) | 0.89 |
| Paradoxical contraction on push | 17/37 (46%) | 10/19 (53%) | 0.78 |
| RAIR present on 50 ml – yes | 18/29 (62%) | 8/17 (47%) | 0.37 |
| Rectal pressure on RAIR (50 ml) – mmHg (n=45) | 142.4 (44.5) | 132.4 (58.4) | 0.11 |
| Rectal pressure on RAIR (50 ml) over 100 mmHg | 23/28 (82%) | 10/17 (59%) | 0.16 |
| BET and sensation performed | 16/37 (43%) | 13/19 (68%) | 0.09 |
| First sensation – ml (n=33) | 40.5 (21.4) | 66.9 (73.9) | 0.04 |
| Urge sensation – ml (n=29) | 58.8 (25.5) | 83.9 (70.9) | 0.15 |
| Maximal tolerated volume- ml (n=26) | 80.7 (33.6) | 115.0 (70.3) | 0.18 |
| Balloon expulsion test – able under 60 seconds | 6/15 (40%) | 4/9 (44%) | 1 |
| Performed BF - yes | 24 (30%) | 5 (14%) | 0.057 |
| Months from surgery to BF (n=25) | 21.7 (19.9) | 25.8 (23.1) | 0.34 |
